# Supplementary material for: Sources of Resilience in Frontline Health Professionals during COVID-19
Source: Healthcare (Basel). 2021 Dec 8;9(12):1699. doi: 10.3390/healthcare9121699 (PMC8702205; doi:10.3390/healthcare9121699)
Supplement: Supplementary file 1 [file healthcare-09-01699-s001.zip › healthcare-1475669-supplementary.pdf]

Guiding Interview Questions.

1. Can you tell me a bit about what it has been like working through the pandemic, and especially some of the changes you have faced at work from before the pandemic to now?

   - Sample follow-up questions: *Have you had to work in a different hospital? Have you transitioned patient groups, for instance from working in elective surgery to working with aged care patients? Have you worked with more patients with advanced care directives in place? Have you had to wear more PPE? Have boundaries between work and home life changed?* How has your worked changed throughout the pandemic? (*first wave with few patients, reprieve, 2nd wave in Victoria with admission of patients)*

2. What have been some of the key challenges during the pandemic? (For example challenges at work at the level of the ward, hospital, organization or industry, or challenges in other environments)

3. Now I want to shift gears and talk about some of the things that have helped you get through this challenging time. What has helped you adjust to the changes and challenges you have been facing?

   a. Have you found any lifestyle factors or routines that have helped you manage during this time?

   b. Are there ways in which other people have helped you get through this?
      i. At work?
      ii. Outside of work? (i.e. friends and family)

   c. What have you found within yourself that has given you the strength to get through this?
      i. Do you think your mindset has helped you get through this challenging time?
      ii. What personal strengths have you drawn on to help you navigate challenges?

4. Have there been any benefits or silver linings for you personally or professionally, working in healthcare during the pandemic?

5. Looking back on the last 6 months, what do you remember the most clearly about the pandemic?
